# The Effect of Zirconium Dioxide (ZrO_2_) Nanoparticles Addition on the Mechanical Parameters of Polymethyl Methacrylate (PMMA): A Systematic Review and Meta-Analysis of Experimental Studies

**DOI:** 10.3390/polym14051047

**Published:** 2022-03-06

**Authors:** Kamila Chęcińska, Maciej Chęciński, Maciej Sikora, Zuzanna Nowak, Sławomir Karwan, Dariusz Chlubek

**Affiliations:** 1 Department of Glass Technology and Amorphous Coatings, Faculty of Materials Science and Ceramics, AGH University of Science and Technology, Mickiewicza 30, 30-059 Krakow, Poland; kamila@checinscy.pl; 2 Department of Oral Surgery, Preventive Medicine Center, Komorowskiego 12, 30-106 Kraków, Poland; maciej@checinscy.pl; 3 Department of Temporomandibular Disorders, Medical University of Silesia in Katowice, Traugutta 2, 41-800 Zabrze, Poland; zuzannaewanowak@gmail.com; 4 Department of Maxillofacial Surgery, Hospital of the Ministry of Interior, Wojska Polskiego 51, 25-375 Kielce, Poland; sikora-maciej@wp.pl; 5 Department of Biochemistry and Medical Chemistry, Pomeranian Medical University, Powstańców Wielkopolskich 72, 70-111 Szczecin, Poland; 6 Department of Maxillofacial Surgery, Regional Specialized Children’s Hospital, Żołnierska 18a, 10-561 Olsztyn, Poland; slawek.karwan@gmail.com

**Keywords:** zirconium dioxide, nanomaterials, polymethyl methacrylate, oral surgery, prosthodontics, PMMA

## Abstract

The number of studies on the subject of effects of zirconium dioxide (ZrO_2_) nanoparticles addition on the mechanical parameters of polymethyl methacrylate (PMMA) is still very limited. Therefore, in this research, the authors wanted to assess PMMA modified with the nano-ZrO_2_ additive in terms of changes in flexural, impact and tensile strength values in relation to PMMA without such component. A systematic review and meta-analysis were performed to evaluate the effect of incorporating nano-ZrO_2_ into PMMA on individual types of material strength. The obtained numerical data were tabulated and analyzed in the search for percentage changes in those parameters. It was then calculated for each set and the procured model was examined using residual sum of squares (RSS) to assess the discrepancy between the data and the estimation model whilst mean absolute deviation (MAD) was employed to determine robustness. The results of the systematic review were composed of data obtained from individual studies presented in eight independent articles. Overall, the addition of nano-ZrO_2_ increases the flexural strength of the composite with the PMMA matrix depending on the size of the ZrO_2_ grains administered. Unfortunately, these conclusions are based on a very limited amount of research and require further verification, especially regarding tensile strength.

## 1. Introduction

The material commonly known as acrylic, is in fact a polymer called polymethyl methacrylate (PMMA). The basic applications of PMMA in dentistry include production of removable dentures, orthodontic appliances and temporarily cemented restorations [1]. PMMA splints, blocks and instruments are fundamental, although not the most prominent, accessories used in maxillofacial surgery [2,3]. They are exerted, among others, for the conservative treatment of fractures, bone positioning during orthognathic procedures, as well as in the treatment of temporomandibular joint dysfunctions [2,4,5]. In some cases, especially regarding management of injuries, it is possible to utilize the patient’s own dentures made of PMMA for maxillofacial surgery. Another application method for acrylic material can be seen in the production of prostheses that restore facial skeleton defects [6]. In this indication, a more extensive than conventional intraoral prostheses and epitheses are put to use [6].

Various types of splints have been implemented in the osteosynthesis of mandible and maxilla fractures. The stabilization of the fragments is achieved by supporting the acrylic on: (1) the gingiva, as in the case of a Gunning splint; (2) gingiva and teeth, as in a Weber splint; (3) teeth only, as in the case of an acrylic block for positioning mandibular condyle fractures. The commonly developed jaw osteosynthesis techniques do not exclude the use of the abovementioned splints. Nevertheless, the introduction of splints in the treatment of fractures has been marginalized and has become applicable in cases where, for various reasons, surgical treatment was abandoned in favor of conservative techniques.

Predesigned orthognathic procedures require intraoperative reference points [3,4,5]. Among these, 3D prints and classic acrylic occlusal splints are quite common [3,4,5,7,8]. In cases of bimax operations, one or two splints can be used at different stages of surgery [8,9]. Whilst the initial splint utilized during the operation is usually quite thick and therefore solid, the second one is composed of a thin layer that positions the jaws in their desired orientation [8,10]. During surgery, strongly pressing the dental arches that were separated by the splint, against each other, is a routine approach that will expose them to damage [8]. In addition, a second, thinner splint, often remains in the patient’s mouth after surgery, which further exposes it to the loads generated by the chewing muscles [8].

Another indication for the use of acrylic in maxillofacial surgery is the treatment of temporomandibular joint dysfunctions [11]. PMMA splints allows for the masticatory muscles’ relaxation, TMJ’s internal derangements can be tended to by maintaining the correct occlusion, increase in vertical occlusal dimension and protecting the prosthetic restorations during and after the reconstruction of the missing teeth [11,12,13,14,15,16]. The types and designs of occlusal appliances are varied due to the differences in their implementation. The classification of occlusal appliances according to Okeson includes: (1) muscle relaxation appliances/stabilization appliances used to reduce muscle activity; (2) anterior repositioning appliances/orthopedic repositioning appliances; (3) anterior bite planes; (4) pivoting appliances; (5) soft/resilient appliances [15]. In turn, classification of occlusal appliances according to Dawson includes (1) permissive splints/muscle deprogrammers; (2) directive splints/nonpermissive splints; (3) pseudopermissive splints [16]. Hard acrylic occlusal appliances are currently favored over soft splints with their superiority confirmed in multiple clinical trials [17,18]. Splint therapy is often substituted or combined with pharmacotherapy, physiotherapy, intra-articular or intramuscular injections [19,20,21]. 

Despite the widespread use of PMMA, this material is easily damaged due to its poor mechanical properties [22,23]. Some attempts have been made to improve the conditions of PMMA’s polymerization and at the same time prosthetic restorations cut from prefabricated blocks are simultaneously prefabricated using computer-aided design and computer-aided manufacturing (CAD-CAM) methods [1]. Recent studies have shown that mechanical properties of CAD-CAM PMMA are better than those of a heat-polymerized PMMA [1].

Another possibility of ameliorating the mechanical properties of PMMA is the use of additives, which result in acquiring composites. The effects implementing additives such as zirconium dioxide nanoparticles (nano-ZrO_2_), titanium dioxide nanoparticles, silicon oxide, aluminum oxide and E-glass fiber have already been studied [23,24]. They resulted, inter alia, in a flexural strength improvement in certain ranges of filler levels and an upgrade in hardness when a particular concentration of fillers was exceeded [24]. With regard to the nano-ZrO_2_ filler, similar quantities, specific to increasing flexural strength, stem from further studies [22,25]. The use of nano-ZrO_2_ as an additive to denture repair materials has already been assessed as promising in a systematic review [23].

Considering that the subject of possible toxicity of nanoparticles is currently undergoing research, the reports that nano-ZrO_2_ additives may have antioxidant and anticarcinogenic effects seem auspicious [26]. There are also studies concerning the development of nano-ZrO_2_ bone substitute materials, where the evaluation of this additive turned out positive [27]. Therefore, we noticed the need to compile and analyze studies on the use of the PMMA/nano-ZrO_2_ composite as the basic material for the fabrication of the abovementioned types of appliances.

## 2. Aim

The aim of this work is to assess PMMA modified with the nano-ZrO_2_ additive in terms of changes in flexural, impact and tensile strength values in relation to PMMA without such an additive.

The primary research objective of this review is to identify the optimal weight concentrations and particle sizes of nano-ZrO_2_ to achieve the highest flexural, impact and tensile strengths of PMMA based composite, which may contribute to the production of more durable appliances in the future. A secondary research objective is to assess the scope of control of the mechanical properties of the PMMA-nano-ZrO_2_ composite by changing the concentration and grain size of the filler, as the most optimal material for a particular application may require a compromise between the values of the abovementioned and other (e.g., thermal) properties.

## 3. Materials and Methods

A systematic review and meta-analysis were performed to evaluate the effect of nano-ZrO_2_ addition to PMMA on individual types of material strength. For this purpose, a search of the records accumulated in medical databases using the PubMed, Bielefeld Academic Search Engine (BASE) and Google Scholar engines was conducted on 7 April 2021. The search strategies were based on the material name, its application related to maxillofacial surgery, the addition of nano-ZrO_2_ and inclusion in the study of any type of changes regarding the durability. These search strategies were initially developed by two authors (M.C. and Z.N.) and then refined and approved by all. The search strategies that were used are presented in Table 1.

For the purpose of determining the articles’ eligibility, the following PICOS inclusion criteria were introduced: (1) Problem—all kinds of splints, acrylic blocks, dentures and other intraoral appliances made of PMMA with the addition of nano-ZrO_2_; (2) Intervention—performance of any type of tests allowing the determination or estimation of at least one of the following: flexural, impact or tensile strengths of the abovementioned appliances; (3) Comparison—as a control sample, it was required to test matching appliances made of PMMA under the same conditions without the addition of nano-ZrO_2_; (4) Outcome—as a result, it was required to provide the flexural/impact/tensile strength values for PMMA material with and without the addition of nano-ZrO_2_; (5) Study design—experimental studies were allowed [28]. Tests performed on repaired appliances were excluded due to the materials’ inhomogeneity. In addition, nonoriginal and non-English-language articles were adopted as exclusion guidelines. A brief summary of the PICOS criteria is provided in Table 2 [28].

The eligibility of the articles found in medical databases was determined using the PRISMA protocol [29]. In the first step, PubMed, BASE and Google Scholar search results were compiled and all duplicates were removed [30]. Then the articles underwent independent screening by two authors (M.C. and Z.N.). Invariably, a single reason for rejecting the article was indicated, i.e., in the case of multiple coexisting reasons, only the first one was stated in accordance with the PICOS criteria sequence [28]. Cohen’s kappa coefficient was used to calculate the compliance of both screening authors’ assessments. Each respective article unanimously qualified by the reviewers as well as all those with a possible conflict were admitted to the full-text eligibility stage. Similar steps were taken at the full-text analysis stage. However, this time it was carried out by a team of three authors (M.C., Z.N. and K.C.) in order to decide by the majority in the event of conflict. Due to an expected limited number of articles meeting the inclusion criteria above, it was decided not to assess the risk of bias.

The results of the systematic review were composed of data obtained from individual studies presented in independent articles. Among which, the sizes of particles and the nano-filler concentration, as well as the values of individual mechanical strengths for the tested PMMA type without additives and with the inclusion of nano-ZrO_2_, were inserted. The obtained numerical data were tabulated and quantitatively analyzed in the search for percentage changes in flexural, impact and tensile strengths parameters. For each data set, the percent change was calculated as the quotient of the end value and the start value of the data. The procured model was inspected using residual sum of squares (RSS) to assess the discrepancy between the data and the estimation model, which is linear regression. Mean absolute deviation (MAD) was used to determine robustness. The extraction, compilation and analysis of data was carried out by two cooperating authors (K.C. and M.C.). In the next stage, the key relationships are presented graphically by one of the authors (K.C.). For this purpose, the OriginLab software (Northampton, MA, USA) was used. The data were then submitted in graphs along with the trend lines and their equations, residual sums of squares, Pearson’s r coefficients and standard errors.

This systematic review is based on the PRISMA 2020 Checklist and PRISMA 2020 for Abstracts [29].

## 4. Results

**Figure 1:**
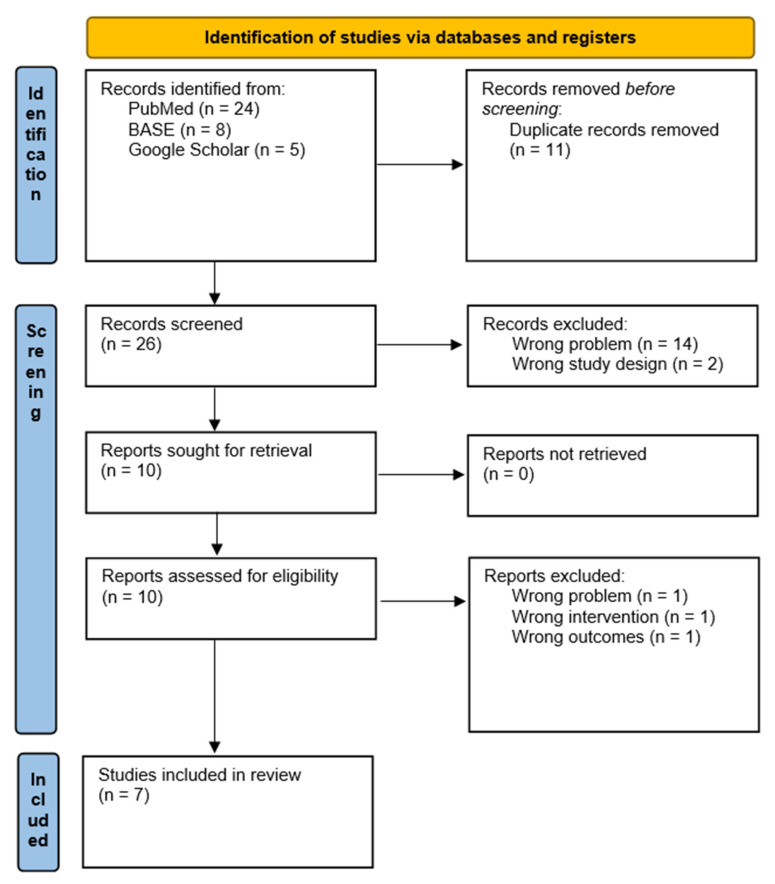
PRISMA flowchart used for the qualification of the studies [29].

In total, 37 relevant records were identified in the three medical databases mentioned before, of which 11 turned out to be duplicates. Amongst 26 unique forms that qualified for screening, a total of 16 were rejected. Cohen’s kappa coefficient at the blind screening stage was k = 0.82 (p_0_ = 0.92; p_e_ = 0.55). In the further course of articles’ qualification, the decisions of all three qualifying reviewers (M.C., Z.N. and K.C.) were unanimous. Out of 10 full-text articles analyzed, 3 had to be rejected. In the first one, studies on more complex materials were carried out, in the second, micro- (not nano-) particles were used whilst in the third, the results obtained did not provide the strength values and therefore could not be compared with other authors’ findings. Additionally, in compliance with the previous assumptions, only 1 qualified article met the assumed PICOS [28] criteria, but it was already identified at the stage of data preparation. Therefore, this article was eventually not included in the PRISMA flowchart (Figure 1) [29]. As a result, this systematic review conclusively selected 8 articles that were fit for the meta-analysis. The content of these 8 articles identified 24 separate studies conducted for different concentrations of nanofiller. These 24 studies were treated individually, each time the average value achieved by the researchers was taken into account (Table 3).

In the entirety of the research, a nanofiller with a grain size of up to 100 nm was used. Additive concentrations of 1 to 20 percent by weight were tested. Thermal polymerization was the common feature of all the evaluated PMMA-ZrO_2_ composites. The most studies collected pertained to the flexural strength trait, which was examined by five authors in 17 screenings. In the cases of impact and tensile strength, four and six tests, that were performed invariably by two teams of authors, were analyzed, respectively. Additionally, it should be mentioned that in the article from 2019, Zidane also studied the impact strength, but in a way that does not allow the inclusion of his work’s results in this analysis [25].

### 4.1. Flexural Strength

**Figure 2:**
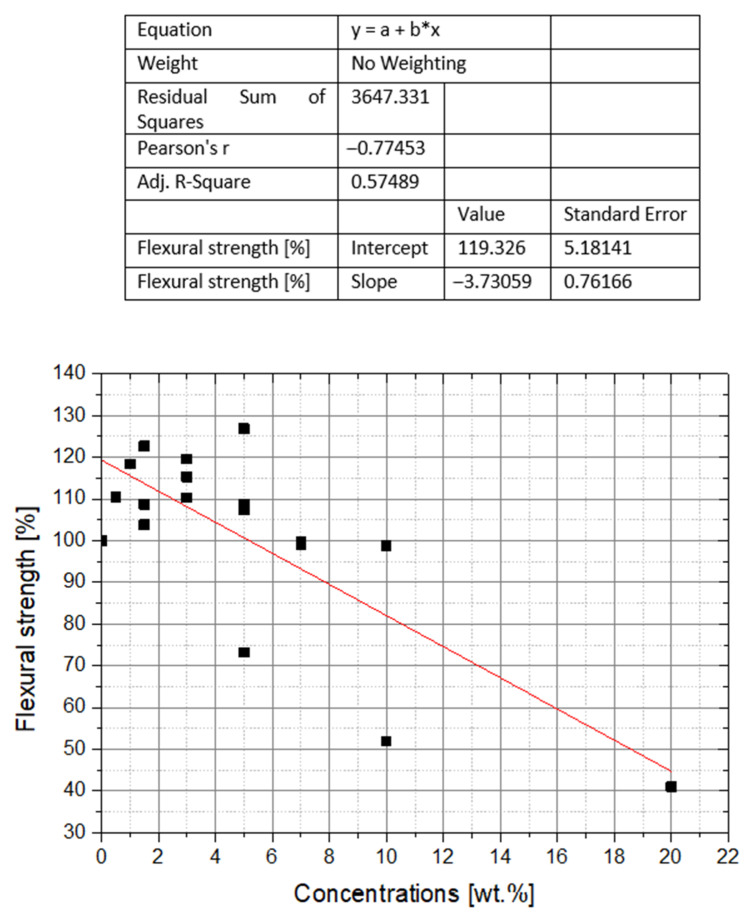
The effect of nano-ZrO_2_ additive concentration on the flexural strength of PMMA composites. The results of individual studies.

**Figure 3:**
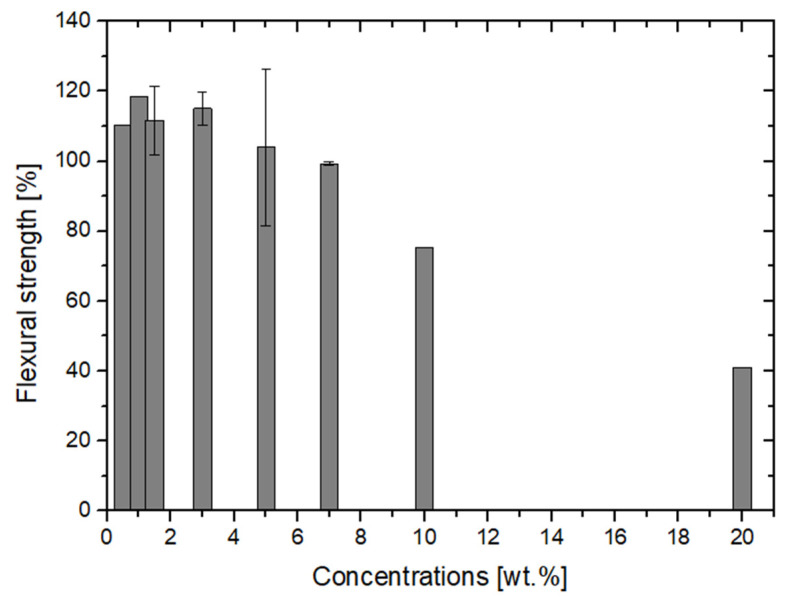
The effect of nano-ZrO_2_ additive concentration on the flexural strength of PMMA composites. The average results for concentrations with variations.

The most abundant data on the PMMA/nano-ZrO_2_ composite durability, concern the flexural strength. In the tested material, the increase in flexural strength was observed in the range from 0.5 to 5 wt% of nanofiller. Nanofiller additions of 7 wt% did not seem to affect the flexural strength. Beyond this value, deterioration of that particular mechanical property of composite is observed (Figure 2 and Figure 3). A high RSS coefficient value for the entire interval, indicates a large discrepancy of the overall data series, and thus a low linear regression fit. On the other hand, the low MAD coefficient (MAD = 0.09) indicates a small spread of data, namely their grouping for the ranges up to 7 wt% and 99–127% flexural strength.

**Figure 4:**
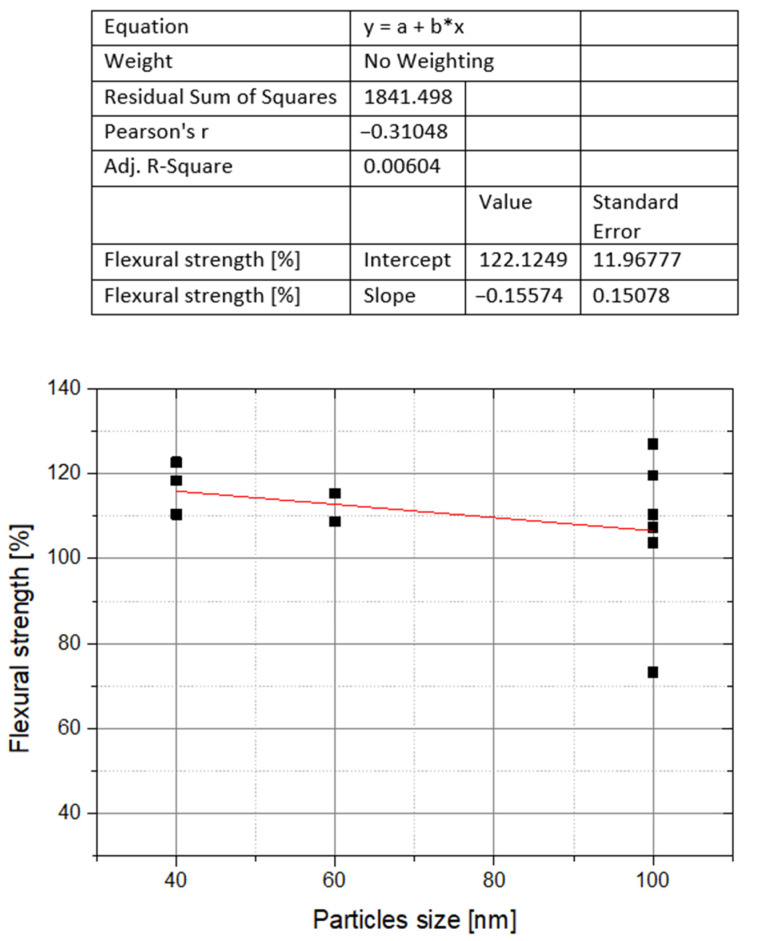
The effect of nano-ZrO_2_ particle size on the flexural strength of PMMA composites for the concentration range of nano-ZrO_2_ from 0.5 to 5 wt%. The results of individual studies.

Taking into account the results of the nano-ZrO_2_ weight concentration influence on flexural strength, the impact of nanofiller particle size was assessed for the concentration range from 0 to 5 wt%. In the studied range, the authors used a nanofiller with grains from 30–40 nm or did not specify the minimum grain size. Therefore, the analysis covered the upper grain size limits, which varied significantly and ranged from 40 to 100 nm depending on the test. The relationship between the upper limit of the nanofiller grain size and the flexural strength of the composite is presented in the graphs (Figure 4). There were no significant differences between the flexural strength values for different maximum grain sizes. In this case, the fit of the data to the estimation model in the form of linear regression is clearly higher, i.e., expressed by a lower RSS coefficient. Nevertheless, a clear limitation of this estimation are the calculations for a large range of grain sizes (40–100 nm) and only three of their values, i.e., 40, 60, and 100 nm.

### 4.2. Impact Strength

**Figure 5:**
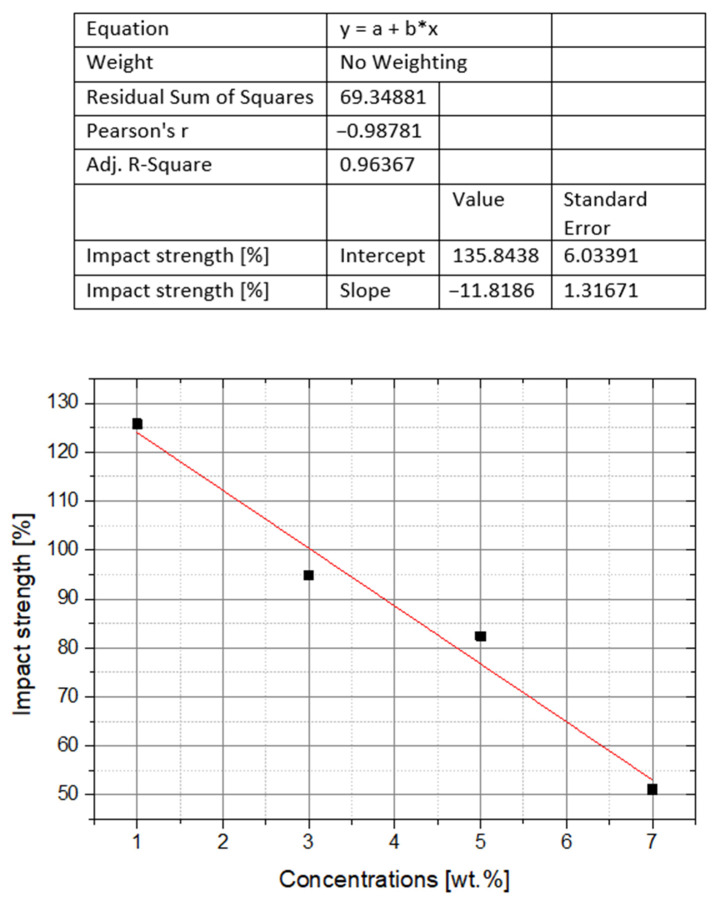
The effect of nano-ZrO_2_ additive concentration on the impact strength of PMMA composites. The results of individual studies.

Only Soundarya et al. demonstrated an increase in impact strength as a result of adding nanofiller to PMMA [35]. It reckoned 26% on average in relation to the control samples for the filler grain size from 30 to 50 nm and 1 wt% [35]. Begum, using the same grain sizes, but higher concentrations from 3 to 7 wt%, achieved only a decrease in impact strength, with a slight reduction, only 5% for the lowest concentration, i.e., 3 wt% [31]. The common size of the grains used in the research of both authors allowed for the graphic presentation of the weight concentrations’ influence on the impact strength (Figure 5) [31,35]. These data are characterized by a good estimation model fit, expressed by a low RSS coefficient value. The Pearson correlation coefficient in this case is nearing one, which proves an almost linear relationship between the data. In turn, the negative value of this coefficient indicates that the decreasing values of the impact strength change are inversely proportional to the increasing values of nanofiller weight concentrations. In this case, the MAD coefficient has a high rate and expresses a large dispersion of data, which is an additional confirmation of the already discussed linear relationship.

### 4.3. Tensile Strength

Tensile strength data are based on two noncomparable studies [32,34]. The study by Ergun et al. was carried out on the basis of nanofiller particles with sizes up to 100 nm, while Gad et al. used nanofiller particles with grain sizes of 40 nm [32,34]. The first authors demonstrated a decrease in tensile strength against PMMA without filler [32]. The characteristic of this decline, depending on the weight concentration of nanofillers, is difficult to assess in this case. On the other hand, Gad et al. presented a relationship close to the linear increase in tensile strength with the rise in nanofiller concentration in the range from 2.5 to 7.5 wt%. For both data series, the MAD value was (MAD = 0.18) and, due to the scarce data, it is difficult to comment on.

## 5. Discussion

### 5.1. General Interpretation of the Results

The use of nanoparticle additives for strengthening the bases of dental prostheses is now widely discussed [23,24,35,36,37]. A recent systematic review by Gad et al., showed an improvement in the repaired dentures’ mechanics, thanks to the use of nano-ZrO_2_, nano-SiO_2_ and nano-Al_2_O_3_ [23]. The aforementioned systematic review took into account the flexural, impact, and tensile strength values assessed in the personal research of other authors [23]. The promising outcome presented by Gad et al. for the cases of prostheses’ repairs prompted the search for general trends in the properties of PMMA matrix materials with the ZrO_2_ nanofiller analyzed in this study [23]. The general interpretation of our systematic review’s results is consistent with other evaluations discussing the effect of nanofillers on the properties of PMMA-based composites [23,38]. More authors agree that nanofillers, including ZrO_2_, may improve the mechanical properties of composites, but only in specific weight concentration ranges of additives with the right grain size [23,25,38].

In order to modify the polymer matrix with nanoparticles, ZrO_2_ is treated with a silane coupling agent. This process is necessary due to the difference in the surface energy of the polymer matrix and the filler. This divergence is a consequence of the hydrophobic nature of the polymer matrix surface and the hydrophilic nature of the filler. The coupling agent used, resulted in the functionalization of the filler surface, and thus improved the bonds at the interface between fillers and matrix [39]. 

The analysis of changes in flexural strength depending on the filler’s weight concentration exhibits that with the increase in the concentration of the modifying phase, the flexural strength of materials deteriorates. This is most likely caused by an uneven distribution of nanofillers in the matrix, and thus their aggregation. This phenomenon is characteristic for particles of this size. This is due to the large surface area of the filler, its chemical activity and high surface energy [40]. The following aggregates can act as stress accumulation centers, and therefore have a negative impact on the material’s mechanical properties [40,41]. Low filler concentrations (up to 5 wt%) have a positive effect on flexural strength due to the possibility of even distribution in the polymer matrix. A large surface area of the filler increases the number of contact points between ZrO_2_ and PMMA, resulting in a confirmed effective interaction at the interface [41]. As a consequence, the nanoparticles take over the stresses loaded on the composite and dissipate them. This process is made possible by stable interfacial bonds and this was achieved through the use of a coupling agent [39].

Additionally, attention should be paid to the influence of particle size on the mechanical properties of materials. Smaller molecules have the opportunity to evenly distribute between the polymer chains, restricting the segmental movements of the macromolecular chains and thus blocking the possibility of their motion, which can be observed by improving flexural strength [42]. 

Ergun et al. obtained lower flexural strength values for various concentrations and particle sizes [32]. This could have probably been caused by specific conditions to which the materials were subjected. The flexural strength test specimens were stored in distilled water for 50 ± 2 h at 37 °C. The flexural strength reduction effect is likely due to tetragonal-to-monoclinic phase transformation of ZrO_2_ [43]. During this process, ZrO_2_ changed its volume causing microcracks in the silane layer [43]. This resulted in the weakening or breaking of the bonds at the PMMA-ZrO_2_ nanoparticles’ shield interface. In addition, through the formed microcracks, water penetrated the polymer network, reducing the flexural strength of nanocomposite [44]. Nevertheless, the flexural strength values acquired for these samples are progressively lower for even bigger nanoparticle concentrations, confirming the conclusions drawn for other materials.

The incorporation of nano-ZrO_2_ also affects the thermal properties as the filler has a higher thermal conductivity than the base material [45,46,47]. Therefore, the thermal conductivity of a composite changes with increasing weight concentration of filler [45,46,47]. Abd Alwahab et al. explain the increase in thermal conductivity of the composite by the role of filler particles as centers of cross-links between PMMA chains [46]. This relationship is not linear, and in the study by Al-Hamadani et al. the highest value of thermal conductivity was achieved for 1 wt% nano-ZrO_2_ additive [45]. Al-Hamadani et al. also showed that the glass transition temperature (Tg) of the composite shifts towards higher temperatures along with an increase in the weight concentration of the nanoadditive [45].

### 5.2. Limitations

The main limitation of this article is the inability to provide a fully up-to-date information. In recent years, there has been progressively more research on enriching PMMA with nanoadditives. Since March 2020, the authors of this systematic review have researched the databases of these articles several times, which resulted in an increasing number of findings. It was decided to establish this review on the final search from April 2021, and since then, only the following stages of the article’s preparation were addressed. For the purposes of defining the limitations of this work, we are obliged to inform the reader that at the time of the last corrections before submitting the manuscript for publication, i.e., in February 2022, the same, very specific, queries for PubMed and BASE gave 29 and 8 records, respectively, which speaks for the need for a permanent tracking progress in this area. The number of BASE records being lower by one compared to December 2021 is due to the specificity of this engine and the gradual reduction of the number of duplicates. The current BASE search actually gives three unique records, which with duplicates is eight records. Among the reports published during the processing of this manuscript, we have identified three that should be considered in subsequent reviews [48,49,50].

Another limitation of this systematic review is the focus on the mechanical properties of composites and the omission of the biocompatibility aspect. At this point, the authors of this work emphasize with full force that the use of nanoadditives in materials that were intended to come into contact with human tissues also requires research regarding their safety of use. Nanoparticles, while promising in terms of material mechanics, may prove inappropriate for their intended applications. To be able to determine this, studies utilizing imitation tissue fluid on cell lines, and then on tissues and living organisms, are needed.

### 5.3. Implications

The addition of nano-ZrO_2_ to PMMA in order to improve the mechanical properties of the composite, relative to that of the pure polymer, may be important in some of the current clinical applications. At present, the use of thin PMMA appliances that are subject to high forces, is taking place in maxillofacial surgery. New composites can be used in the future to fabricate occlusal plates for orthognathics and splints used in maxillary and mandibular fractures. A new perspective, not yet available for PMMA-based materials, is the fabrication of durable crowns and prosthetic bridges as long-term restorations.

## 6. Conclusions

The addition of nano-ZrO_2_ with values from 0.5 to 5 wt% increases the flexural strength of the composite with the PMMA matrix. The ZrO_2_ concentration by weight seems to have a directly proportional effect on the impact strength, giving the best result for a concentration of 1 wt%. The size of the ZrO_2_ grains used may affect the flexural strength; better results were obtained for smaller grains. We were unable to draw tensile strength results due to insufficient data. All of the above conclusions are based on a very limited number of studies and require further verification.

## 7. Other Information

### Registration and Protocol

This systematic review of experimental studies has not been registered, as currently only systematic reviews based on human or animal studies are subject to registration in accordance with the PRISMA guidelines in the PROSPERO database [29]. The protocol of this systematic review has not been prepared as a separate article and has not been previously published.

## Figures and Tables

**Table 1:** Search strategies used for identification of studies.

Search Strategy	
PubMed	(PMMA OR acrylic) AND (splint OR splints OR prosthesis OR prostheses OR denture OR dentures) AND (zirconium OR zirconia OR ZrO_2_) AND (nano OR nanoparticles) AND strength
BASE	Title: (PMMA acrylic) AND (splint splints prosthesis prostheses denture dentures) AND (zirconium zirconia ZrO_2_) AND (nano nanoparticles) AND strength
Google Scholar	allintitle: (PMMA OR acrylic) (splint OR splints OR prosthesis OR prostheses OR denture OR dentures) (zirconium OR zirconia OR ZrO_2_) (nano OR nanoparticles) strength

**Table 2:** A brief summary of the PICOS criteria used for determining the eligibility of the studies [28].

	Inclusion Criteria	Exclusion Criteria
Problem	Appliances made of PMMA with nano-ZrO_2_ additive	Repaired appliances
Intervention	Flexural strength and/or impact strength and/or tensile strength tests	-
Comparison	Appliances made of PMMA without additives	-
Outcome	Flexural strength and/or impact strength and/or tensile strength assessments	-
Study design	In vitro studies	Non-original and/or non-English papers

**Table 3:** Summary of the studies qualified for the meta-analysis. For individual types of durability, the percentage strength in relation to the reference value obtained in the test for PMMA without nanofiller is given.

First Author, Publication Year	Nano-ZrO_2_ Particle Size, nm (Average)	Nano-ZrO_2_ Concentration, wt%	Flexural Strength	Impact Strength	Tensile Strength
Alhotan, 2021 [24]	<100	1.5	104%	-	-
		3.0	110%	-	-
		5.0	107%	-	-
		7.0	99%	-	-
Begum, 2019 [31]	30–50	3.0	-	95%	-
		5.0	-	82%	-
		7.0	-	51%	-
Ergun, 2018 [32]	<100	5.0	73%	-	93%
		10.0	52%	-	96%
		20.0	41%	-	87%
Gad, 2020 [33]	40	0.5	110%	-	-
		1.0	118%	-	-
		1.5	123%	-	-
Gad, 2018 [34]	40	2.5	-	-	123%
		5.0	-	-	128%
		7.5	-	-	134%
Soundarya, 2021 [35]	30–50	1.0	-	126%	-
Zidan, 2020 [22]	30–100	3.0	120%	-	-
		5.0	127%	-	-
Zidan, 2019 [25]	30–60	1.5	109%	-	-
		3.0	115%	-	-
		5.0	109%	-	-
		7.0	100%	-	-
		10.0	99%	-	-

## Data Availability

All collected data is available in this article.
